# A synergistic approach towards understanding the functional significance of dopamine receptor interactions

**DOI:** 10.1186/1750-2187-8-13

**Published:** 2013-12-05

**Authors:** Pratima Pandey, Mahlet D Mersha, Harbinder S Dhillon

**Affiliations:** 1 Department of Biological Sciences, Delaware State University, Mohali 140308, India; 2 Department of Biological Sciences, Center for Neuroscience Research, Delaware State University, Dover, DE 19901, USA

**Keywords:** Dopamine receptor, G-protein, GPCR, G*α*, *Caenorhabditis elegans*

## Abstract

The importance of the neurotransmitter dopamine (DA) in the nervous system is underscored by its role in a wide variety of physiological and neural functions in both vertebrates and invertebrates. Binding of dopamine to its membrane receptors initiates precise signaling cascades that result in specific cellular responses. Dopamine receptors belong to a super-family of G-protein coupled receptors (GPCRs) that are characterized by seven trans-membrane domains. In mammals, five dopamine receptors have been identified which are grouped into two different categories D1- and D2-like receptors. The interactions of DA receptors with other proteins including specific Gα subunits are critical in deciding the fate of downstream molecular events carried out by effector proteins. In this mini-review we provide a synopsis of known protein-protein interactions of DA receptors and a perspective on the potential synergistic utility of *Caenorhabditis elegans* as a model eukaryote with a comparatively simpler nervous system to gain insight on the neuronal and behavioral consequences of the receptor interactions.

## Background

Dopamine (DA) is a catecholamine neurotransmitter that plays a central role in nervous system development and its apt function later in life. DA is known to regulate many neuronal and physiological activities ranging from movement control to cognition, emotion, positive reinforcement, food intake, and endocrinal regulation. In mammals, cell bodies of dopaminergic neurons originate from the ventral tegmental area (VTA) of the midbrain extending processes to the cortex and limbic areas of forebrain to establish the mesocortical and mesolimbic pathways reviewed in [1]. Dysregulation of these pathways can lead to pathological states including Parkinson’s disease, Huntington’s disease, schizophrenia, psychoses, and attention deficit hyperactivity disorder reviewed in [2]. In addition, a number of socio-behavioral disorders such as alcoholism, drug addiction, and depression are also suggested to be based on DA dysfunction reviewed in [3]. As with most other complex neuronal pathways, dopamine cross talks with other neurotransmitters and a subset of traditional dopaminergic neuronal subpopulations within VTA may also co-transmit the excitatory neurotransmitter glutamate along with dopamine reviewed in [4]. Availability of a number of comprehensive reviews including those referenced above, on various aspects of dopaminergic transmission underscore the enormous effort by researchers on uncovering the functional dynamics of this key neurotransmitter. Readers are cautioned that this mini-review restricts its perspective on the utility of *Caenorhabditis elegans*, a model eukaryote with a simple nervous system and well-defined neural connectivity to synergize investigations into the biological relevance of molecular interactions of DA receptors, and is not intended as a comprehensive structural-functional review of dopamine triggered signaling. The worm model, offers phenomenal level of genetic accessibility, knowledge of neuronal connectivity circuits and behavioral assays. Studies in a whole organism can be useful in understanding the functional consequences of the DA receptor interactions as well as in developing potential therapeutic targets.

Precise molecular interactions determine the specificity and efficacy of dopaminergic signaling pathways, which are largely conserved in both invertebrates and vertebrates The effect of DA is transduced through cell surface G-protein coupled receptors (GPCRs) that are characterized by seven transmembrane domains and based on pharmacological profiles and binding specificity to hetero-trimeric G-proteins, they are classified as D1-like or D2-like receptors [5,6]. G-proteins consist of α, β and γ subunits and binding of DA to its receptor causes a GDP to GTP exchange in the α subunit resulting in its activation and release from the βγ heterodimer so as to recruit downstream effectors [7]. D1-like receptors signal by coupling to Gαs (stimulatory G-protein) and closely related Gα_olf_ proteins. D2-like receptor signaling is mediated through inhibitory, Gαi/o class of G-proteins that are sensitive to pertussis toxin. Studies in the last two decades have shown that besides adenylate cyclase, there are various other effectors including ion channels, archidonic acid, mitogen activated protein kinases (MAPKs), sodium proton exchangers that can be activated by dopamine receptors [8,9]. In addition, investigators have unearthed several proteins, such as calcium-binding proteins, cytoskeletal proteins, chaperones and endoplasmic reticulum associated proteins and the more common Gα subunits, which can directly interact with D1- and D2-like receptors [9]. Here, an overview of known protein-protein interactions of dopamine receptors is provided with a focus on the utility of a model eukaryote with a comparatively simpler nervous system *Caenorhabditis elegans* to integrate understanding of cellular dopaminergic signaling with behavioral correlates at the whole organism level.

*C. elegans* is a eukaryotic experimental model that is widely used in contemporary research in molecular neurobiology due to its simpler nervous system, small genome, and availability of convenient forward and reverse genetic tools. *C. elegans* has been established as a successful model system to investigate various aspects of metabolic, genetic, neurobiological and neurodegenerative diseases prevalent in humans [10]. At a fundamental level, the organism shares cellular and molecular mechanisms with humans. This microscopic nematode worm has ~19,800 predicted protein-coding genes, and the fate of each of the 959 somatic cells present in the adult hermaphrodite has been tracked through development, and the connectivity for all its 302 neurons is known [11,12]. In a *C. elegans* hermaphrodite which is the predominant sex (>99% of wild type population) there are just eight dopaminergic neurons [11]. The completely sequenced *C. elegans* genome reveals that it has homologues for a majority of human genes, and this model organism has helped pioneer the identification of key genes involved in a number of important processes including development, signal transduction, cell death, neural function, and drug discovery [13]. Neuronal studies in *C. elegans* are favorable due to high-resolution live imaging of neurons in its transparent body and the development of various behavioral assays including development of automated worm tracking programs that allow the simultaneous monitoring of different behaviors including their endo-phenotypes [14,15].

### Dynamics of dopamine signaling via G-protein coupling

Mammalian model systems have provided a broader understanding of dopamine signaling which can be divided into three major stages: (i) activation of dopamine receptors at the cell surface by ligands, (ii) interaction of receptor with heterotrimeric G-proteins, and (iii) signal transduction through effector molecules to generate cellular responses. Dopamine receptors transduce signals by coupling to G-proteins, composed of α, β and γ subunits. There are 20 known Gα subunits, grouped into 4 subfamilies (Gαs, Gαi, Gαq, and Gα12), 5 Gβ subunits and 12 Gγ subunits which participate in a wide range of cellular activities from development to signaling [16]. D1-like receptors transduce signals by coupling to Gαs (stimulatory G-protein) and a closely related Gα_olf_ (G-protein involved in olfaction), which lead to activation of adenylyl cyclase (AC), resulting in an increase in cAMP production. D2-like receptor signaling is mediated through inhibitory Gα_i/o_ class of G-proteins which are sensitive to pertussis toxin and cause inhibition of adenylate cyclase activity. Adenylate cyclase further activates Protein Kinase A (PKA) that results in the phosphorylation of specific downstream effector molecules some of which influence gene expression [6,17,18]. Both D1-like and D2-like receptors can also alternatively couple to other Gα proteins and Gβγ subunits. This coupling of D1-like or D2-like dopamine receptors to a diverse group of G-proteins can occur simultaneously or alternately [18].

**Figure 1:**
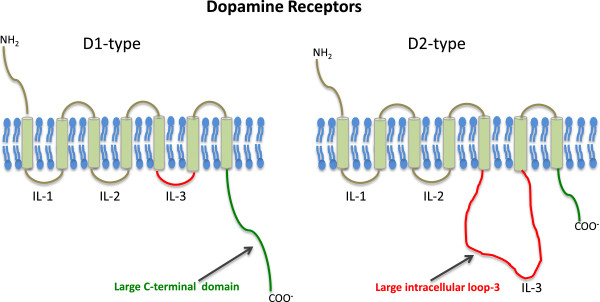
Schematic of structures highlighting comparative general differences between D1- and D2-like dopamine receptors.

Dopamine receptors like other GPCR proteins are characterized by 3 intracellular loops (il_1-3_), 3 extracellular loops (ol_1-3_), an extracellular amino terminus (N_t_) and cytoplasmic C terminal (C_t_) tail [19]. While the transmembrane regions are generally conserved amongst the GPCRs, there are variations in other regions of these proteins. Comparison between D1-like and D2-like receptors reveals that C terminal (C_t_) tail in D1-like receptors is significantly longer as compared to D2-like receptors (Figure 1). On the other hand, D1-like receptors have short third intracellular loop (il3) whereas D2-like receptors are characterized by a long third intracellular loop [20,21]. Multiple isoforms of mammalian receptors exist *in vivo*. Isoforms of D2-like receptors are generated due to alternative splicing of the pre-mRNA in the region coding for their third intracellular loop. Two isoforms of human D1 receptor are known and result from alternate use of transcriptional initiation sites. The human D2 receptor is known to have 3 isoforms, Short, D2-Long, and D2-Longer [5,22]. The presence of splice variants for these receptors provides isoforms with a wider range of choices to couple to G-proteins. The diversity of dopamine receptor subtypes, along with the heterotrimeric nature of the G-proteins themselves, provides key components for complex signaling networks within the neuronal cell. A number of studies indicate that the third intracellular loop of DA receptors is essential for G-protein coupling and specific regions of interaction lie near the N- and C-terminal regions of the loop [23-27]. It remains to be determined if other regions of the receptor facilitate or actively participate in G-protein coupling. Additionally, the mechanism by which a receptor can selectively discriminate between closely related subtypes of G-proteins remains unclear or whether such coupling is dictated by the receptor itself. Furthermore, several aspects of receptor-G protein coupling remain unresolved. These include deducing reliable consensus sequences on the receptor for each G-protein subtype. Investigations in these directions can help unveil mechanisms governing how signals are transmitted and fine-tuned, within cells through regulation of DA receptors.

### Dopamine receptor protein-protein interactions

Receptors are amongst the primary targets for drugs, and the need to identify dopamine receptors’ interacting proteins that are likely to directly influence dopaminergic pathways remains fundamentally important to assess their suitability as therapeutic targets. DA receptors can interact with various proteins either through their extracellular loops, intracellular loops or C-terminal regions. Their intracellular interactions are known to occur with a variety of proteins including ion channels, cytoskeleton proteins, signaling proteins, scaffolding and adapter proteins. Even though, in general, D2-receptors act antagonistically to D1 receptors and inhibit cAMP activity, they may also show synergistic effects when co-expressed in the same neurons where they may form hetero-oligomers [28,29]. Together, D1 and D2 receptors in mammals are found to induce calcium release that is distinct from D1 or D2 receptor pathways activated independently [30-32]. It has been suggested that unusual or excessive formation of D1-D2 hetero-dimer complexes may play a part in major depression in humans, and uncoupling of such complexes in the rat model can have antidepressant effects [33]. Dopamine receptors can also crosstalk to molecular pathways initiated by other receptors or with scaffold proteins which then help integrate the two pathways. Proteins known to interact with D1- and D2-like receptors include: a chaperone protein calnexin [34], DRiP78 (Dopamine receptor-interacting protein 78) [35], calcium binding proteins S100B, cytoskeletal proteins 4.1 N [36], arrestin 2 and arrestin 3 [23]. Arrestins are important trafficking proteins that help in receptor desensitization and re-sensitization during GPCR signaling. After being exposed to continued or repeated stimulation with an agonist, receptor desensitization may occur. During desensitization, agonist induced activation of receptors is followed by receptor phosphorylation through GPCR kinases (GRKs) or Protein Kinase A [37]. Arrestins are also known to act as a scaffolding protein, promoting the stable association of signaling proteins with the receptor. Phosphorylated receptors are bound to arrestins or other interacting proteins that terminate GPCR signaling, resulting in rapid desensitization of the receptor by inhibiting receptor binding to G-proteins. Arrestins can also target receptors to clathrin-coated pits for internalization and degradation, thereby preventing re-sensitization [38,39]. Protein-protein associations of DA receptors have been suggested to determine the expression of receptors at the cell surface and maintain their density. Several cytoskeletal proteins (filamin A, protein 4.1 N and actin binding protein-ABP280) and an endoplasmic reticulum protein (calnexin) interact with D1/D2 receptors and control surface expression and receptor signaling [34,36,40]. Calcium binding proteins S100B and calmodulin bind to dopamine receptor D2 to regulate the activation of effector ERK signaling pathway [41]. D2 receptor also interacts with spinophilin (also called neurabin) that links the receptors and various downstream molecules to the actin cytoskeleton, thus regulating signaling within neurons that receive dopaminergic input [42]. A comprehensive list of known specific proteins that interact with mammalian dopamine receptors is provided along with their cellular function, and their *C. elegans* counterparts’ are included with the mutant phenotype of the latter (Table 1).

**Table 1:** Cellular function of mammalian proteins known to interact with DA receptors and the mutant phenotypes of their worm homologues

**Mammalian DA receptor interacting proteins**		***C. elegans *** **orthologues**	
**Protein**	**Function [Reference]**	**Orthologues**	**Mutant phenotype [Reference]**
Calcium binding protein S100B	Increased D2 receptor stimulation of extracellular signal-regulated kinases and inhibition of adenylate cyclase [41].	CNB-1	*cnb-1* mutants show thinner cuticle, decreased brood size and delayed egg laying [43]*tax-6* mutants are defective in thermosensation [44]. The specific role of CAL-1 is not known.
		CAL-1	
		CNA-1 (TAX-6)	
CaM Kinase II	Phosphorylation by CaMKII sensitizes D3 receptor [45].	UNC-43	*unc-43* mutation lengthens the period of the motor output as well as reduces locomotor activity [46-48].
NCS-1	Desensitization of D2 receptor through calcium sensing [49].	NCS-1	*ncs-1* knockout animals show deficits in isothermal tracking behavior [50].
Arrestin 2	Desensitization through inhibition of D2 receptor-G-protein interaction [23].	ARR-1	*arr-1* mutants are defective in olfactory adaptation [51,52].
ER protein Calnexin	Trafficking of D1 and D2 receptors to the cell surface [34].	CNX-1	At 25°C *cnx-1* mutants show increased lethality, slow growth, and lower brood size [53].
Protein 4.1 N	Cell surface expression and co-localization and stability of D2 receptor subtypes [54].	FRM-4	Not known
		FRM-10	
Spinophilin (Neurabin)	Provides scaffold for D2 receptors and relay molecules [42].	NAB-1	*nab-1* mutants display reduced synapse density and resistance to paralysis on aldicarb [55].
Neurofilament M	Regulates D1 cell surface expression, and receptor desensitization [56].	Not known	Not known
Filamin- A	Adaptor for linking D2-like receptors with cytoskeletal actin [40,57].	FLN-1	*fln-1* mutants have defective spermatheca and reduced brood size [58].
Paralemmin	Attenuation of D2 mediated as well as receptor-independent generation of cAMP [59].	LMN-1	*lmn-1* mutants show major deficits due to abnormal condensation of chromatin, abnormal distribution of nuclear pore complexes and chromosome loss in some cells [60].
CLIC6	Interaction with D2 receptors potentially regulates chloride channel [61].	EXC-4	The tubular excretory cell lumen in *exc-4* mutants is disrupted by swellings similar to cysts found in tubulocystic kidney disease [62,63].
β-catenin	D2 receptors’ interaction with β-catenin inhibits wnt/calcium signaling pathway [64].	SYS-1	*sys-4* mutants display gonadogenesis defects [65].
NCAM-180	Internalization of D2 receptors [66].	NCAM-1	Not known
Transient receptor potential channel TRPC1	D2 interaction enhances TRP1 delivery to cell surface [67].	TRP-4	*trp-4* mutants are defective in proprioception and mechanosensation [68,69].
GIPC	Attenuates D2 signaling through regulator of G-protein RGS19 [70].	C35D10.2	Not known
Gamma COP	Transport of D2 receptors to neuronal membrane [71].	T14G10.5	RNAi knock-down of T14G10.5 causes defects in locomotion and reduced fertility [72].
DRiP78	Transport of D1 receptors from ER to neuronal membrane [35].	DNJ-5	Not known

It is emerging that dopamine receptors like other GPCRs are dynamic complexes, that involve interactions between receptor–receptor, receptor–G-protein and receptor-interacting proteins, all of which help control the intricate and finely tuned process of signal propagation. A consequence of the complexity of dopaminergic signaling is that there remain significant gaps in understanding the basic organization of GPCRs and their effectors, and therefore, there is limited understanding of precise role of dopaminergic signaling pathways in neurological diseases. Dopamine signaling studies done via ectopic expression in heterogenous cell lines have been very informative, however it is important to recognize that they do not recapitulate the multifaceted milieu provided by a functional nervous system inside an organism. Thus utilizing synergistic information obtained through a whole organism model such as *Caenorhabditis elegans* in analogous studies can be advantageous. This model eukaryote overcomes the isolated environment of *in vitro* or cellular models and allows the study of complex neural mechanisms in an intact native environment. This “whole animal system” provides the additional advantage of tracking actual behavioral responses in the animal.

### Dopamine receptor function in *C. elegans*

In *C. elegans* dopamine regulates neuronal functions and participates in a wide array of nematode behaviors such as locomotion, food sensation, egg laying, defecation, learning and memory (Table 1) [73-80]. Primarily, studies with this organism have utilized either reduction in dopamine synthesis by laser ablation of dopaminergic neurons, genetic mutants, and pharmacological antagonists or enhancement of dopamine levels by providing exogenous dopamine or agonists. In the hermaphrodite worm, dopamine is synthesized in eight neurons: two anterior deirid neurons (ADEs), two posterior deirid neurons (PDEs) and four cephalic neurons (CEPs) while an additional six dopaminergic neurons are also located in the male tail [11,81,82]. Four dopamine receptors have been characterized in *C. elegans* and two additional receptor genes have been recently curated in the worm genome. Based on its sequence profile and pharmacological properties DOP-1 is classified as a D1-like receptor [83], DOP-2 and DOP-3 are D2-like receptors, while DOP-4 is an invertebrate specific receptor [77,84,85]. Unlike mammalian D1-like receptor isoforms that result from alternate transcriptional initiation, the four known DOP-1 variants in worms result from alternate splicing [77,86,87]. D2-like receptors in both mammals and *C. elegans* have two or more splice variants with differences limited primarily to the lengths of their third intracellular loops [84,88]. However DOP-4 does not have any splice variants. DOP-1 mutant studies have elucidated its role in locomotion, basal slowing and habituation response to tap stimulus [77,78,89]. The DOP-2 auto-receptor has been reported to modulate dopamine release in associative learning [79]. DOP-3 works antagonistic to the DOP-1 mediated physiological processes and signaling [90,91]. DOP-3 and DOP-4 plays a role in avoidance response to aversive soluble repellents [92,93]. Two newly curated open reading frames namely T02E9.3 and C24A8.1 represent *dop-5* and *dop-6* and the gene product of the latter may act redundantly with DOP-2 [94,95]. Table 2 summarizes known interactions of the *C. elegans* dopamine receptors along with their known roles in behavior. Additional details on specific mutant allele phenotypes, expression patterns and pharmacological profiles of DA receptors are available [85].

**Table 2:** **A summary of known effectors of *C. elegans *** **dopamine receptors and their physiological functions**

**Receptor**	**G-proteins**	**Effectors**	**Physiological role**
DOP-1	EGL-30/ Gαq	ITR-1, PKC-1, EGL-8	Tap habituation, locomotion, acetylcholine release [77,78,90,91].
DOP-2	GPA-14, GOA-1	Unknown	Associative learning, anterior touch habituation, suppression of octopamine-mediated CREB activation [32,78,79,88].
DOP-3	GOA-1/ G_αo/i_	DAG kinase (DGK-1)	Enhancement of 2-nonanone avoidance, negative regulation of locomotion, and octanol hyper-sensitization [90,92,96].
		RGS protein (EAT-16)	
		RGS-3	
DOP-4	Not known	Not known	General enhancement of avoidance responses [93].

The *C. elegans* genome encodes for 21 G*α*, 2G*β* and 2 G*γ* proteins. Four of the Gα genes *goa-1*, *gsa-1*, *egl-30*, *gpa-12* belong to mammalian classes of Gα family G*α*_i/o_, G*s*, G*q* and G*12,* respectively. The remaining 17 G*α* genes are closer in sequence similarity to G*α*i/o proteins but display limited similarity to the mammalian classes [97,98]. The G*α* proteins in *C. elegans* also interact with dopamine receptors to transmit signal upon activation. G*αq* encoded by EGL-30 was shown to mediate dopamine signaling through DA receptor DOP-1 and DOP-3 signaling is mediated through G_o_ protein GOA-1 [78,90,91]. These two pathways function antagonistically in *C. elegans* and has been suggested to explain the functional effects of analogous pathways in mammalian brain that regulate neurotransmitter release by working in a manner opposite to each other [91]. Dopamine regulates various aspects of behavior in *C. elegans* which has been investigated using mutants in the individual DA receptors: *dop-1* mutants are deficient in habituation, a simple form of non-associative learning [77,78] and *dop-2* mutants are defective in habituation as well as associative learning. These abnormalities are rescued by the application of exogenous dopamine [79,80]. Upon encountering food, worms display basal slowing, which is a decrease in their rate of locomotion. Loss of function mutants of the D2-like receptor *dop-3* display abnormal basal slowing responses, and mutations in *dop-1* have been shown to rescue this defective response [90-98]. In addition, *dop-3* deletion mutants have abnormal octanol avoidance responses (62). DOP-4 enhances repellant responses towards copper and this deficit is not rescued by providing exogenous dopamine [93]. With the exception of DOP-1, the remaining worm dopamine receptors have been shown to be involved in amphetamine (AMPH) dependent behaviors such as swimming induced paralysis (SWIP), when worms were placed in water [99]. Interestingly, the transcription factor HLH-17 influences the expression of *dop-*1, *dop-2* and *dop-3,* and *hlh-17* mutants display deficits in behaviors requiring a functional dopaminergic signaling [100]. It has been suggested that another transcription factor CREB known for its role in long-term memory, is modulated antagonistically by DOP-1 and DOP-3 [91,101]. In summary, studies with *C. elegans* have helped bridge the gap between dopamine function at a molecular and cellular level on one hand, and the neuronal output affecting behavior on the other.

## Conclusions

The importance of dopamine receptors in neuronal signaling has led to numerous elegant studies that have produced an enormous amount of valuable information relevant to the molecular signaling pathways used by the D1- and D2- receptor types. The vast and sometimes contradictory results from cell culture systems, combined with the complexity of the pathways involved has inspired many researchers to study the precise role of dopamine receptors with a holistic perspective. Since the dopamine receptor interacting proteins are conserved between vertebrates and invertebrates therefore, investigating the functional role of dopamine receptors and their interacting proteins in a whole organism such as *C. elegans* provides a contiguous “molecule to behavior” *in vivo* system. Towards this end, other basic biomedical research models including fruit flies, zebra fish and mice also offer similar platforms aimed at the functional dissection of dopamine receptors’ interactions in terms of their *in vivo* and overt biological consequences. The advantage of *C. elegans* is that its behavior is controlled by a limited set of neurons whose connections can be precisely traced to provide fundamental insights in understanding complex processes in higher organisms. Greater insight into the precision and subtleties involved in dopamine receptor regulation from a simple yet complete system, will eventually require extrapolation to mammalian systems in the future.

## Abbreviations

GPCRs: G-protein coupled receptors; G-protein: Guanine nucleotide-binding protein; Gαs: Stimulatory G-alpha subunit; Gαi: Inhibitory G-protein.

## Competing interests

The authors declare that they have no competing interests.

## Authors’ contributions

All authors (PP, MDM and HSD) contributed synergistically to conceive, write, update and review the manuscript, and approve the final manuscript.

## Authors’ information

PP is a Research Scientist at the Indian Institute of Scientific Research and Education, MDM is a Graduate student at Delaware State University and HSD is an Associate Professor of Neuroscience at Delaware State University.
